# Antimicrobial stewardship programme in Sri Lanka: insights from early implementation experience (2024–2025)

**DOI:** 10.1016/j.lansea.2026.100826

**Published:** 2026-07-27

**Authors:** Malika Karunaratne, Priyantha Lakmini Athapattu, Melanie Naamal Jayawardena, Chathuri Gunasekera, Deepika Abeywarna Arachchi, Lilani Karunanayake, Kushlani Jayatilleke, Samiddhi Samarakoon, Kumara Wickramasinghe, Ananda Wijewickrama, Upul Dissanayake, Suranga Manilgama, Mohamed Nawzar Ayisha Nusaiha, Chathuranga Lakmal Fonseka

**Affiliations:** a Sri Lanka College of Microbiologists, Colombo, Sri Lanka; b National Institute of Infectious Diseases, Angoda, Sri Lanka; c National Focal Point for Antimicrobial Resistance, Ministry of Health, Colombo, Sri Lanka; d Ministry of Health, Colombo, Sri Lanka; e National Cancer Institute of Sri Lanka, Maharagama, Sri Lanka; f Department of Medical Microbiology & Immunology, Faculty of Medicine, University of Colombo, Colombo, Sri Lanka; g Base Hospital, Tangalle, Sri Lanka; h Medical Research Institute, Colombo, Sri Lanka; i Sri Jayewardenepura General Hospital, Nugegoda, Sri Lanka; j National Hospital of Sri Lanka, Colombo, Sri Lanka; k College of Medical Administrators of Sri Lanka, Colombo, Sri Lanka; l Sri Lanka Medical Association, Colombo, Sri Lanka; m Ceylon College of Physicians, Rajagiriya, Sri Lanka; n Sri Lanka College of Internal Medicine, Colombo, Sri Lanka; o Faculty of Medicine, University of Ruhuna, Galle, Sri Lanka; p Department of Medicine, Faculty of Medicine, University of Ruhuna, Galle, Sri Lanka

**Keywords:** Antimicrobial resistance, Antimicrobial stewardship, Developing countries, Low-cost implementation framework, Strategic plan implementation

## Abstract

Bridging the gap between national policy and hospital-level implementation remains a critical challenge for antimicrobial stewardship (AMS) in low- and middle-income countries (LMICs). This health policy outlines the phased rollout of Sri Lanka’s centrally mandated National AMS programme (2024). Driven by the Ministry of Health and Sri Lanka College of Microbiologists, the intervention deployed four standardised, low-cost tools: a national institutional framework, a localised WHO AWaRe classification, a dedicated antimicrobial prescription chart, and revised empirical guidelines. To overcome resource constraints, the strategy utilised a ‘Training-of-Trainers' model and multisectoral coalitions across all nine provinces. A one-year post-launch structural evaluation demonstrated an implementation rate exceeding 50% across all key performance indicators. Integration of the AWaRe classification achieved the highest uptake (74%), while establishing functional AMS teams and Point prevalence surveys achieved 52%. Sri Lanka’s experience demonstrates that centralised governance and pragmatic, low-cost structural tools can institutionalise sustainable AMS frameworks in resource-constrained settings.

## Introduction

Antimicrobial resistance (AMR) is a leading global health threat, disproportionately affecting low- and middle-income countries (LMICs).^1^^,^^2^ Despite Sri Lanka’s robust and free public healthcare system,^3^ unregulated access and suboptimal antimicrobial prescribing practices remain critical challenges. The public healthcare system of Sri Lanka comprises a total of 1208 facilities distributed across three tiers: tertiary care (55 National, Teaching, Specialised, and District General Hospitals), secondary care (71 Base Hospitals), and primary care (Divisional Hospitals and Primary Medical Care Units). Both public and private healthcare sectors allow direct patient access to care without mandatory referrals. This model drives unregulated antimicrobial dispensing and exacerbates community-level prescribing challenges. Furthermore, local evidence reveals a critical gap between physician knowledge and actual prescribing practices.^4^ Across outpatient, inpatient, and critical care settings, studies consistently document pervasive empiric overprescription, poor documentation of clinical indication and a lack of routine antimicrobial reviews.^5^ Among outpatients over one-third received antibiotics (primarily for respiratory symptoms), while 45% of inpatients were prescribed antimicrobials and one-third among these were reported to be inappropriate.^6^^,^^7^ Consequently, Sri Lanka faces higher resistance rates; national Global Antimicrobial Resistance and Use Surveillance System (GLASS) data indicates meropenem resistance reaches 11% in *Escherichia coli and* 48% in *Klebsiella pneumoniae* in blood culture isolates, while resistance to front-line agents is four to six times higher than in high-income countries.^8^^,^^9^ This is also expected to cost Sri Lanka an estimated USD 96.2 million annually, highlighting that effective national stewardship could avert over a billion dollars in projected GDP losses.^10^^,^^11^

Prior to the current national initiative, Sri Lankan AMS activities were largely fragmented, and lacked a standardised ward-level framework. Although high-level governance existed, including an AMR focal point, a national advisory committee, the National Strategic Plan (2017–2022),^12^ and a 28-site resistance surveillance network reporting to WHO GLASS; clinical translation was hindered by lack of accountability. A 2016 'Red Light’ circular mandated microbiologist pre-authorisation for broad-spectrum agents but lacked a functional implementation mechanism. Furthermore, the shortage of microbiologists in secondary and some tertiary centres limited oversight. Consequently, stewardship relied on isolated awareness programs.

In response, the Ministry of Health (MoH) launched the second National Strategic Plan (2023–2028), aligning with WHO’s Global Action Plan under a “One Health” framework.^13^^,^^14^ To address one of the seven priority areas, namely rational antibiotic use, the MoH and Sri Lanka College of Microbiologists (SLCM) designed a systematic, hospital-based national antimicrobial stewardship (AMS) programme across all nine provinces, guided by the WHO toolkit for resource-limited settings.^15^ This health policy aimed to describe the design and implementation of Phase one of AMS programme and outlines three focal areas: (1) the development & identification of pragmatic, low-cost national AMS tools tailored for resource-constrained settings; (2) the systematic deployment of these interventions through a centrally driven programme and cascade training model; and (3) the early implementation outputs evaluating the feasibility, and uptake of the programme across most tertiary care and few secondary care hospitals.

This national-scale implementation study was conducted across Sri Lanka’s public healthcare system. While the national health infrastructure comprises primary, secondary, and tertiary care facilities, this initial Phase 1 rollout utilised purposive sampling to establish a robust foundational network. All tertiary care hospitals at the time were selected including three national hospitals, 12 teaching hospitals, 12 specialised hospitals, and 18 district general hospitals (DGH’s). Nine of eighteen DGHs did not have microbiology specialist access while one had an off-site microbiologist. Further, five base hospitals with microbiology specialty access were also included.

**Fig. 1:**
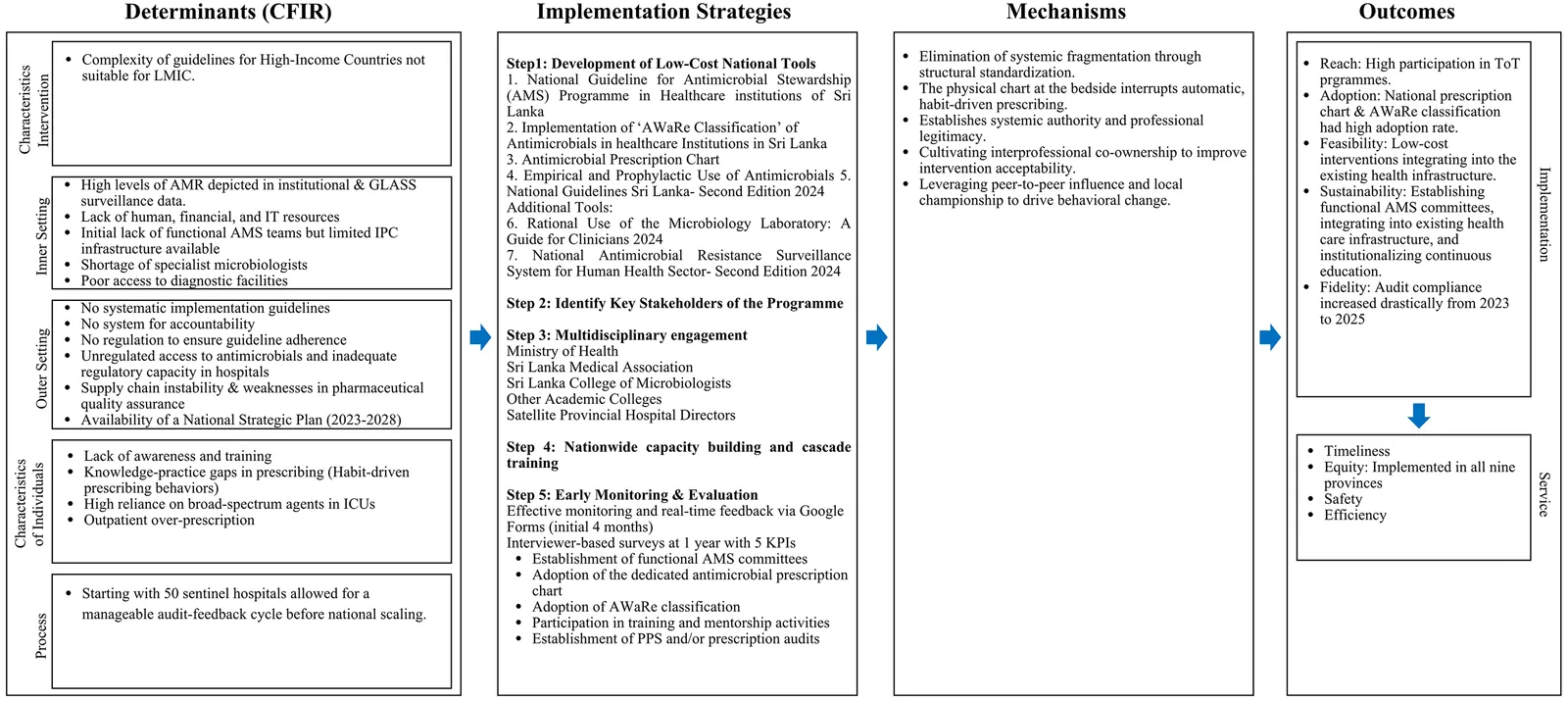
Implementation research logic model of the phase 1 of antimicrobial stewardship programme in Sri Lanka.

The implementation aligned with Consolidated Framework for Implementation Research (CFIR) framework. To systematically evaluate the programme’s theory of change, the project architecture linked contextual barriers to pragmatic interventions, their mechanisms of action and multi-level outcomes. We retrospectively applied the Implementation Research Logic Model (IRLM) (Fig. 1) to deconstruct the implementation process and evaluate the relationships between contextual barriers and observed outcomes.^16^ The intervention was modelled on successful global stewardship frameworks,^13^^,^^15^^,^^17^^,^^18^ specifically adapted to the existing MoH administrative structure and deployed through five strategic interventions (Step 1–5). The programme's timeframe is outlined in Supplementary Table S1.

## Development of low-cost national AMS tool

Four low-cost national AMS tools and a supplementary tool were developed by SLCM technical working groups aligning with the NSP for AMR and WHO stewardship principles.^17^ These tools are summarised in Fig. 1. They include (i) guidelines for implementation of AMS across institutions; (ii) regulation of ‘Access’, ‘Watch’, and ‘Reserve’ antimicrobial use through graded prescribing authority and mandatory microbiology authorisation for reserve agents to address irrational broad-spectrum initiation^19^; (iii) the Antimicrobial Prescription Chart that was introduced in 2024 to mandate documentation of clinical indications, allergy status, pre-treatment cultures, and AWaRe categorisation, which importantly, institutionalises clinical reviews at 48 h, 7 days, and 10 days, interrupting automated prescribing habits and streamlining audit data capture for prescription audits and Point prevalence surveys (PPS); and (iv) a guideline for standardised antimicrobial regimens was also introduced prioritising narrow-spectrum antimicrobials.^20^^,^^21^^,^^22^ Additionally, a supplementary tool was developed to align AMR surveillance protocol with GLASS requirements,^9^ which is aimed at improving understanding of local epidemiology of AMS. These tools underwent rigorous review including pilot studies and multidisciplinary input from the professional bodies. Each tool was institutionalised by a MoH circular or as a national guideline recognising them as mandatory implementation guides. Further, these tools were also distributed to private sector healthcare institutions through Private Health Sector Regulatory Council under the MoH.

## Identifying key stakeholders of the programme

Implementation was led by the SLCM as the technical and coordinating body, while the MoH provided the necessary regulatory authority and funding. A centralised structure was deliberately selected over decentralised stewardship models that rely on individual hospitals to independently build and implement their own programs. This top-down approach utilised uniform guidelines to empower administrators and ensure equitable implementation across hospitals despite resource disparities like lack of on-site microbiologists.

## Mobilising multidisciplinary engagement & awareness

The collaborative strategy secured the institutional support necessary for systemic integration, and ensured the interventions were practical. Through consensus-building, from professional endorsement for national guidelines to pragmatic co-designing for daily operational tools and training modules, professional bodies and front-end users became active stakeholders instead of passive recipients. To ensure sustainable implementation, institutional buy-in was established through direct collaboration with local hospital administrators. Furthermore, official authorisation of the programme across the health system via government circulars was achieved by the Director General of Health Services (DGHS) through the AMR focal point.

To maximise reach, protocols were disseminated through established, high-visibility platforms. Tools were introduced to hospital administrators during monthly directors’ meeting chaired by the Director General of Health Services. Advocacy efforts were amplified through Continuing Medical Education (CME), expert panel discussions and World Antimicrobial Resistance Awareness Week (WAAW) campaigns.

## Nationwide capacity building and cascade training

To operationalise the four main AMS national tools, a centralised cascade training strategy was implemented. To ensure high attendance, the Ministry of Health (MoH) issued official invitations with paid duty leave and incentivised participation with Continuous Professional Development (CPD) points. The SLCM experts developed a 6-h face-to-face Training-of-Trainers (ToT) module including pre- and post-training assessments, structured lectures incorporating behaviour change components, facilitated small-group activities designed to provide practical feedback through different SWOT-analysis scenarios, and hands-on exercises for accurately completing the newly introduced antimicrobial prescription chart. ToT workshops were conducted for multidisciplinary teams including hospital directors, clinicians, medical officers, nursing leaders, and pharmacists, empowering them as institutional “AMS champions” responsible for implementation and cascade training in their respective hospitals. The simultaneous nationwide rollout leveraged peer influence among hospital directors to reinforce local AMS governance. For sustainability, this capacity-building was integrated into the MoH’s annual ‘Programme for Master Trainers on Healthcare Quality and Safety’ conducted annually by the healthcare quality and patient safety directorate.

Online Stewardship mentorship programme was conducted six months after the islandwide training-of-trainers (ToT) initiative, with support from the WHO South-East Asia Regional Office (SEARO). The programme engaged eighteen hospitals across the public and private sectors to sustain momentum and deepen impact. The hospital teams were trained to be stewardship mentors, and a one-year activity plan was funded by the WHO in participating hospitals.

## Early monitoring of the implementation

During the initial four-month rollout, implementation progress was assessed by utilising Google forms and one-year post-implementation, a cross-sectional baseline survey was conducted. An interviewer-based questionnaire was administered under the guidance of AMR Focal Point, and the data which was verified through direct interviews with consultant microbiologists or hospital directors or AMS team members under five previously identified Key Performance Indicators (KPIs). It includes establishment of functional AMS committees, adoption of the dedicated antimicrobial prescription chart, adoption of AWaRe classification, participation in training and mentorship activities and establishment of point prevalence surveys (PPS) and/or prescription audits. The KPIs are defined in Supplementary Table S2. The main objective of this survey is to assess whether the required structures, systems, and processes have been established for AMS implementation in the 50 targeted hospitals.

The implementation of the National Antimicrobial Stewardship (AMS) programme was evaluated across 50 sentinel hospitals according to defined indicators. The comparative results of the pre- and post-implementation assessments are presented in Table 1.

• Capacity Building: The reach of the ToT programme was assessed by institutional participation in nine provincial workshops conducted in each province. The national Training-of-Trainers (ToT) program achieved 76.8% attendance rate (945/1230 invited professionals). Ten programs were carried out in all 9 provinces (2 in Western). Following, the ToT and implementation of the Antimicrobial Stewardship in phase 1 hospitals, a KPI revealed that 70% (35/50) of hospitals conducted at least one internal awareness and training programme for prescribers, nurses, and pharmacists in 2025 compared to 12% in 2023.
• National Stewardship Tools: Adoption and Penetration of the programme were measured through the utilisation of the WHO AWaRe classification and the National Antimicrobial Prescription Chart.
  ○ AWaRe Classification: 74% (37/50) of hospitals implementing pre-authorisation protocols for at least a selection of ‘Reserve’ group drugs. Notably, out of all tertiary care hospitals in the programme, 15/15 National and Teaching hospitals achieved adoption of this restrictive prescribing strategy.
  ○ Prescription Chart: Implementation rate of the adoption of the dedicated antimicrobial prescription chart was 72% (36/50) in the phase 1 hospitals with either partial or complete adoption.
• PPS and Audit Compliance: These essential practices increased from a baseline of 10% of these hospitals having conducted at least one formal audit or PPS in 2023 to 52% in 2025.
• Institutionalisation of Governance: The sustainability of the intervention was evaluated through the establishment of functional AMS committees, defined as having held at least one formal meeting in 2025. Overall, 52% (26/50) of phase 1 hospitals maintained functional AMS committees.

**Table 1:** Pre- and post-antimicrobial stewardship phase 1 key performance indicators survey results.

Parameter assessed	Pre AMS survey (2023)	Post AMS survey (2025) in phase 1 hospitals	Post AMS survey (2025) in phase 1 hospitals with consultant microbiologist supervision
Presence of functional AMS Teams^a^	5/50 (10%)	26/50 (52%)	26/42 (61%)
Adoption of the dedicated antimicrobial prescription chart^b^	6/50 (12%)	36/50 (72%)	36/42 (85%)
Adoption of AWaRe classification^c^	0/50 (0%)	37/50 (74%)	37/42 (88%)
Staff awareness/ training programmes^d^	6/50 (12%)	36/50 (72%)	36/42 (85%)
Point prevalence surveys^e^	5/50 (10%)	26/50 (52%)	26/42 (61%)

a Functional AMS Teams (At least one team meeting for the year 2025).

b Adoption of the dedicated Antimicrobial Prescription Chart (Assessed whether antimicrobials are prescribed on the dedicated chart) Partial (in several wards) or Complete (all wards).

c Adoption of AWaRe classification (Pre-authorisation for at least a few Reserve Group drugs) AWaRe classification adopted in Sri Lanka in 2024.

d Staff Awareness (At least one awareness programme for the hospital staff annually - prescribers, nurses, pharmacist). Not including WAAW programmes.

e PPS/Audits (Conducted at least one for the year 2025).

In phase 1 hospitals where microbiologists supervision is available, implementation rates were remarkably higher for all indicators in the order of 61%, 85%, 88%, 85% and 61% for the KPIs.

## Discussion

This one-year, Phase 1 rollout across 50 sentinel hospitals, focused on structural foundations of stewardship rather than clinical outcomes. Overall, achieving a baseline implementation rate exceeding 50% across diverse public health facilities demonstrates the feasibility of this low-cost, task-shifting model. The high compliance in the adoption of the AWaRe classification (74%) was bolstered by pre-existing ‘red-light’ restriction protocols. Since literature suggests that prospective audits and feedback constitute the most promising strategy to drive behavioural modification towards appropriate antibiotic use, we used a standardised national prescription chart that embeds cognitive forcing functions into routine clinical care.^23^ Conversely, the formal appointment of AMS committees was the least implemented objective (51%), reflecting a pragmatic adaptation to utilise pre-existing Infection Prevention and Control (IPC) teams for AMS duties. Predictably, structural implementation gaps were most pronounced in District general hospitals lacking specialist microbiologists, and within specialised institutions (e.g., Eye and Dental hospitals) where generic national tools failed to accommodate niche clinical workflows.

Regional evidence reveals varied AMS success across Asia. In Pakistan, structured programmes exist in less than one-third of facilities.^24^ An initiative in Vietnam that used mainly guidelines, reduced total antimicrobial consumption but failed to improve prescribing habits (∼80% inappropriate) underscoring the need for pragmatic tools that physically intervene at the point of care, as seen in the Sri Lankan model.^25^ The Indian Medical Council’s initiative (2019–2021) achieved near-universal compliance in three years but relied on US$15,000 annual funding per hospital for dedicated staff and IT infrastructure.^26^ In contrast, the Sri Lankan programme leveraged existing state supply chains and task-shifting current staff.^25^ While the funded Indian model reached higher compliance (95%), the Sri Lankan framework demonstrates a sustainable, low-cost alternative for establishing structural foundations in resource-limited settings without external financial dependency.

A defining characteristic of this national rollout was its financial feasibility, utilising existing government mechanisms rather than external funding. The interventions circumvented the need for capital-intensive IT infrastructure by relying on established paper-based workflows. Human resource expenditures were minimised through strategic task-shifting, empowering existing multidisciplinary staff as ‘AMS Champions' rather than recruiting dedicated personnel. Furthermore, framework development and ToT facilitation relied on voluntary expertise from professional colleges and WHO consultants. Therefore, the external financial inputs were utilised as targeted, catalytic investments rather than foundational operational costs; for example, WHO SEARO provided one-year grants to twenty mentorship hospitals, upon the development and implementation of localised AMS activity plans to drive sustained institutional momentum. However, due to logistical delay the funds were received only after the one-year cross-sectional survey.

Several limitations must be acknowledged. Facilities lacking resident microbiologists showed lower implementation fidelity (Table 1). While “AMS Champions” mitigated this, their lack of formal authority underscores the need to shift more responsibility to pharmacists and nursing staff. Methodologically, the absence of physical ward audits and the evaluation’s guidance by the National AMR Focal Point risk both social desirability and internal evaluation biases, though independent auditing was pragmatically unfeasible. Contextually, unpredictable shortages of “Access” antibiotics frequently forced clinicians to bypass guidelines. Finally, slow laboratory turnaround times created a “diagnostic-stewardship gap,” forcing prolonged broad-spectrum therapy and highlighting the need to synchronise prescribing tools with laboratory capacity.

To strengthen the current framework and ensure the sustainability of the AMS programme, we propose key strategic priorities:

• Establishment of a national team to oversee digital reporting and conduct rapid audit-feedback cycles to ensure accountability and data transparency.
• Scaling up of activities to secondary, primary, and private healthcare sectors supported by Asian Development Bank and Pandemic Fund grants. Phase II which will commence in 2026 is aimed to focus on secondary care hospitals.
• Mandatory inclusion of AMS and infection prevention control guidelines within professional development curricula of healthcare workers to foster continuous improvement.
• Partnership with general practitioners, pharmacists, and community leaders to curb self-medication and unregulated use.
• Strengthening laboratory supply chains and introduction of rapid detection capacities to support timely, evidence-based prescribing.
• Optimising procurement and regulatory oversight to ensure the availability of quality-assured antimicrobials to prevent stock-outs.

Further longitudinal research is required to correlate these systemic improvements in early implementation outcomes with reductions in resistance rates, healthcare-associated infections, and mortality using long term data. Ultimately, these initiatives should culminate in a centralised digital dashboard for real-time surveillance of consumption and resistance trends, facilitating integrated stewardship.

## Conclusion

Sri Lanka’s Phase 1 rollout demonstrates that a centrally mandated, low-cost AMS framework is feasible and effective in resource-limited settings. By leveraging existing infrastructure and task-shifting through a ‘Training-of-Trainers' model, the programme institutionalised standardised stewardship tools nationwide, achieving substantial structural uptake within one year. While specialist oversight remains a critical success factor, the transition from ad hoc activities to a unified national system provides a sustainable model for other LMICs. Future phases must now focus on digitising monitoring to translate these systemic foundations into measurable clinical improvements.

## Contributors

MK, PA and CLF conceptualised and designed the paper. MK, CLF, MNJ and LK wrote the first draft with substantive inputs to the text from all co-authors. MK led the project administration and resource management with inputs from SS, KW, AW, UD, and SM. PA assisted in funding allocation. MK, MNJ, CG, DAA, LK, KJ, KW, AW, UD, and SM led the policy design work and technical development. CLF collected, compiled, and synthesised bibliographic data. MK, CLF and MNAN managed the editing and reviewing process for the final submission. All authors reviewed the final draft of the initial submission and approved the manuscript.

## Declaration of interests

All authors declare no competing interests.
