# Supplementary material for: Antimicrobial stewardship programme in Sri Lanka: insights from early implementation experience (2024–2025)
Source: Lancet Reg Health Southeast Asia. 2026 Jul 27;53:100826. doi: 10.1016/j.lansea.2026.100826 (PMC13551883; doi:10.1016/j.lansea.2026.100826)
Supplement: Supplementary file 1 [file mmc1.docx]

**Supplementary Table 1: Phase 1 of Antimicrobial Stewardship Programme in Sri Lanka**

| **Topic** | **2024** | | | | | | | | | | | | **2025** | | | | |
| --- | --- | --- | --- | --- | --- | --- | --- | --- | --- | --- | --- | --- | --- | --- | --- | --- | --- |
|  | **Jan** | **Feb** | **March** | **April** | **May** | **June** | **July** | **Aug** | **Sep** | **Oct** | **Nov** | **Dec** | **Jan** | **Feb** | **March** | **Apr** | **Nov** |
| **Conceptualization** | X |  |  |  |  |  |  |  |  |  |  |  |  |  |  |  |  |
| **Step 1:** | | | | | | | | | | | | | | | | | |
| **Development of Antimicrobial stewardship National Guideline & Circular** |  |  | X | X | X | X | X |  |  |  |  |  |  |  |  |  |  |
| **Development of National Antimicrobial Prescription Chart** | X | X | X | X | X | X |  |  |  |  |  |  |  |  |  |  |  |
| **Publication of AWaRe Classification Circular as a tool** |  |  |  | X |  |  |  |  |  |  |  |  |  |  |  |  |  |
| **Launching of Updated National Antimicrobial Guideline** |  |  |  |  |  |  | X |  |  |  |  |  |  |  |  |  |  |
| **Step 2** | | | | | | | | | | | | | | | | | |
| **Identifying Key Stakeholders** | X |  |  |  |  |  |  |  |  |  |  |  |  |  |  |  |  |
| **Step 3** | | | | | | | | | | | | | | | | | |
| **Multisectoral Collaboration** |  | X | X | X | X | X | X | X | X | X | X |  |  |  |  |  |  |
| **Step 4** | | | | | | | | | | | | | | | | | |
| **ET&R fund application** |  |  |  |  | X | X | X |  |  |  |  |  |  |  |  |  |  |
| **ToT Workshop in all nine provinces** |  |  |  |  |  |  | X | X | X | X | X |  |  |  |  |  |  |
| **WHO SEARO Mentorship** |  |  |  |  |  |  |  |  |  |  |  |  | X | X | X | X |  |
| **Step 5** | | | | | | | | | | | | | | | | | |
| **Real-time Feedback from Provincial Focal Points** |  |  |  |  |  |  | X | X | X | X | X | X | X | X |  |  |  |
| **1-year Post Implementation Survey** |  |  |  |  |  |  |  |  |  |  |  |  |  |  |  |  | X |

**Supplementary Table 2: Key-Performance Indicators Definitions**

| KPI | Definition |
| --- | --- |
| Functional AMS Committees | Conducted at least one formal committee meeting in the year 2025. |
| Prescription Chart Adoption | Utilization of the dedicated chart for antimicrobial prescribing, recorded as either complete (all wards) or partial (selected wards). |
| AWaRe Classification Adoption | Active pre-authorization protocols established for at least a selection of "Reserve" and/or "Watch" group antimicrobials. |
| Training & Mentorship | Conducted at least one internal awareness programme annually for hospital staff (prescribers, nurses, pharmacists), excluding WAAW events. |
| Audit Compliance | Conducted at least one Point Prevalence Survey (PPS) or formal prescription audit in the year 2025 to ensure local fidelity. |
